# Changes in salivary biomarkers of stress, inflammation, redox status, and muscle damage due to *Streptococcus suis* infection in pigs

**DOI:** 10.1186/s12917-023-03650-z

**Published:** 2023-07-31

**Authors:** María José López-Martínez, Mario Andre S. Ornelas, Roxana Elena Amarie, Edgar Garcia Manzanilla, Silvia Martínez-Subiela, Fernando Tecles, Asta Tvarijonaviciute, Damián Escribano, Antonio González-Bulnes, José Joaquín Cerón, Marina López-Arjona, Alberto Muñoz-Prieto

**Affiliations:** 1 Interdisciplinary Laboratory of Clinical Analysis (Interlab-UMU), Regional Campus of International Excellence ‘Campus Mare Nostrum, University of Murcia, Espinardo, Murcia, 30100 Spain; 2 Pig Development Department, The Irish Food and Agriculture Authority, Teagasc, Moorepark, Fermoy, Co Cork, P61 C996 Ireland; 3 School of Veterinary Medicine, University College Dublin, Belfield, Dublin, Ireland; 4 Department of Agriculture, Food, and Environment, University of Pisa, Pisa, Italy; 5 Departamento de Producción y Sanidad Animal, Facultad de Veterinaria, Universidad Cardenal Herrera-CEU, CEU Universities, C/Tirant lo Blanc, 7, Alfara del Patriarca, Valencia, 46115 Spain

**Keywords:** Inflammation, Pigs, Redox status, Saliva, Sepsis, *Streptococcus suis*, Stress

## Abstract

**Background:**

*Streptococcus suis* (*S. suis*) is a Gram-positive bacteria that infects pigs causing meningitis, arthritis, pneumonia, or endocarditis. This increases the mortality in pig farms deriving in severe economic losses. The use of saliva as a diagnostic fluid has various advantages compared to blood, especially in pigs. In this study, it was hypothesized that saliva could reflect changes in different biomarkers related to stress, inflammation, redox status, and muscle damage in pigs with *S. suis* infection and that changes in these biomarkers could be related to the severity of the disease.

**Results:**

A total of 56 growing pigs from a farm were selected as infected pigs (n = 28) and healthy pigs (n = 28). Results showed increases in biomarkers related to stress (alpha-amylase and oxytocin), inflammation (haptoglobin, inter-alpha-trypsin inhibitor heavy chain 4 (ITIH4), total protein, S100A8-A9 and S100A12), redox status (advanced oxidation protein producs (AOPP)) and muscle damage (creatine kinase (CK), CK-MB, troponin I, lactate, aspartate aminotransferase, and lactate dehydrogenase). An increase in adenosine deaminase (ADA), procalcitonin, and aldolase in infected animals were also observed, as previously described. The grade of severity of the disease indicated a significant positive correlation with total protein concentrations, aspartate aminotransferase, aldolase, and AOPP.

**Conclusions:**

This report revealed that *S. suis* infection caused variations in analytes related to stress, inflammation, redox status, and muscle damage in the saliva of pigs and these can be considered potential biomarkers for this disease.

## Background

The Gram-positive bacteria *Streptococcus suis* (*S. suis*) is one of the most frequent infectious diseases in pigs producing high mortality and major economic losses [1]. The most common lesions associated are meningitis, arthritis, pneumonia, or endocarditis [2]. This infection is also an important zoonosis, with the number of cases increasing in the last years, especially associated with the ingestion of uncooked pork and inadequate preventive barriers at slaughterhouses [3, 4].

Saliva contains analytes that can be used as biomarkers of various physiological pathways and reactions such as stress, inflammation, or sepsis [5]. It is also a non-invasive easy-to-collect sample that does not induce stress, making saliva a particularly interesting sample in pigs where blood collection is especially traumatic [5, 6], although it also can be used in other farm animals such as ruminants [7].

In a previous report, *S. suis* infection was shown to produce changes in the proteomic profile of saliva, due to activation of selected pathophysiological mechanisms leading to changes in selected analytes, e.g., an increase in adenosine deaminase (ADA), a marker of the immune system [8]. In addition, procalcitonin (PCT) and aldolase, biomarkers of sepsis, have been described to be increased in the saliva of pigs infected by *S. suis* [9]. These three analytes could be considered potential biomarkers for *S. suis* infection [9].

The hypothesis of this investigation is that in saliva of pigs infected with *S. suis* there could be changes in analytes other than those previously described (ADA, PCT, or aldolase) [8–10] and these analytes could be potential biomarkers of this disease. Therefore, the objective of this study was to evaluate the possible changes in different biomarkers of stress, inflammation, redox status, and muscle damage in pigs infected with *S. suis*. For this purpose, a comprehensive panel integrated by analytes that can reflect these different physiological pathways was measured. This panel consisted of cortisol, alpha-amylase (sAA), and oxytocin (OXT) as biomarkers of stress, haptoglobin (Hp), inter-alpha-trypsin inhibitor heavy chain 4 (ITIH4, pig-MAP) and total proteins as indicators of inflammation, and the ferric-reducing ability of saliva (FRAS) and advanced oxidation protein products (AOPP) as biomarkers of redox status. Also, the S100A8-A9 (calprotectin, CALP) and S100A12 (calgranulin C), which are proteins related to inflammation and immunity [11] and also sepsis [12], were analyzed. In addition, creatine kinase (CK), CK-myocardial band (CK-MB), troponin I, lactate, lactate dehydrogenase (LDH), aspartate aminotransferase (AST), and alanine aminotransferase (ALT) were measured to assess possible muscle damage in the saliva of pigs with *S. suis* infection. These analytes were evaluated along with previously described biomarkers that are changed in *S. suis* infection, such as ADA, PCT, and aldolase [8–10].

## Results

### Changes of salivary analytes in pigs with meningitis by *S. suis*

The results (median and range) of the salivary biomarkers are presented in Table 1. In the group of biomarkers related to stress, sAA, cortisol, and oxytocin increased 9, 1.7, and 1.8-fold respectively in pigs with meningitis compared with healthy pigs. The biomarkers of inflammation haptoglobin and ITIH4 showed higher levels in pigs with meningitis than controls (1.5 and 1.6-fold, respectively). The total protein concentration also increased (1.9-fold) in pigs with meningitis with respect to healthy pigs. S100A8-A9 and S100A12 showed increases of 3.4 and 3.6-fold, respectively, in pigs with meningitis compared to healthy animals.

**Table 1:** Variations in the salivary biomarkers of pigs with meningitis (n = 28) compared with the healthy group (n = 28)

	Healthy group		Meningitis group		
	Median	Range	Median	Range	P value
**Stress**					
Alpha-amylase (U/L)	814.4	169.6–3264	7306	848–168,979	< 0.001
Cortisol (ng/mL)	42.40	16.8–105.6	72.4	29.2–212	0.032
Oxytocin (pg/mL)	2256	506–3834	4108	1270–20,000	0.004
**Inflammation**					
Haptoglobin (mg/L)	2.51	0.85–4.23	3.87	3.48–5.86	0.021
ITIH4 (µg/L)	17.70	2.64–43.94	32.96	7.10–519	0.023
Total proteins (mg/dL)	135.9	34.24–288.2	264.4	156.6–316.4	0.001
S1000A8-A9 (Calprotectin) (mg/L)	0.07	0.06–0.3	0.24	0.06–1.62	< 0.001
S100A12 (Calgranulin C) (mg/L)	0.20	0.03–0.92	0.72	0.31–4.72	< 0.001
**Redox status**					
FRAS (µmol/L)	260	80–560	300	140–420	0.516
AOPP (µmol/L)	122	33– 372	190	56–262	0.042
**Muscle damage**					
Creatine kinase (U/L)	89.2	26–184.3	123.5	58.1–787.2	0.011
Creatine kinase – MB (ng/mL)	44.1	12.1–67.7	84.3	22.2–238.1	0.026
Troponin I (ng/mL)	0.24	0.2–0.53	0.44	0.2–2.5	0.014
Lactate (mmol/L)	9.2	1.8–55.2	30.1	5–307	0.042
Lactate dehydrogenase (U/L)	37.8	5.3–267.5	204.9	36.7–771.6	< 0.001
Aspartate aminotransferase (U/L)	111.3	30.6–306.9	287.2	94.9–109.1	< 0.00
Alanine aminotransferase (U/L)	20.2	10.9–68.8	49.7	16.8–77.1	0.023
**Sepsis**					
Procalcitonin (ng/mL)	1857	245.2–4994	6121	30.5–33,736	0.014
Aldolase (U/L)	15.2	8.9–51.2	25.5	13.9–63.8	0.012
**Immune system**					
Adenosine deaminase (U/L)	2008	1750–2688	18,400	7620–75,136	< 0.001

ITIH4: inter-alpha trypsin inhibitor 4; FRAS: ferric reducing ability of saliva; AOPP: advanced oxidative protein products

Among the redox status markers, AOPP showed a significant increased of 1.5-fold in pigs with meningitis. In the case of muscle damage biomarkers, all of them showed higher levels in pigs with meningitis compared with healthy pigs: CK (1.3-fold), CK-MB (1.9-fold), troponin I (1.8-fold), lactate (3.27-fold), LDH (5.4-fold), AST (2.5-fold), and ALT (2.4-fold).

The immune system biomarker ADA increased 9.1-fold in the saliva of pigs with meningitis compared with controls. In addition, PCT and aldolase increased 3.3 and 1.6-fold, respectively, in pigs with meningitis compared with healthy pigs.

The ROC curve analyses showed AUCs higher than 0.8 (Table 2) for sAA (AUC = 0.96), total proteins (AUC = 0.92), S100A18/A9 (AUC = 0.89), S100A12 (AUC = 0.88), LDH (AUC = 0.87), PCT (AUC = 0.86), AST (AUC = 0.84), PCT (AUC = 0.86) and ADA (AUC = 0.92) (Table 3).

**Table 2:** Receiver operating characteristic (ROC) curve analysis for determination of cutoff value for the salivary analytes investigated in pigs with meningitis

	Area under the ROC curve (AUC)	95% CI	P values	Cutoff value	Sensitivity (%)
**Stress**					
Alpha-amylase (U/L)	0.96	0.96-1	< 0.001	2224	95.24
Cortisol (ng/mL)	0.71	0.53–0.88	0.03	46.40	81.25
Oxytocin (pg/mL)	0.79	0.64–0.95	0.005	2071	89.47
**Inflammation**					
Haptoglobin (mg/L)	0.69	0.52–0.86	0.02	2.50	72.73
ITIH4 (µg/L)	0.73	0.56–0.91	0.023	21.23	68.75
Total proteins (mg/dL)	0.92	0.81-1	0.002	199	83.33
S1000A8-A9 (mg/L)	0.89	0.69–0.96	< 0.001	0.175	85
S100A12 (mg/mL)	0.88	0.77–0.99	< 0.001	491.2	83.33
**Redox status**					
FRAS (µmol/L)	0.58	0.33–0.84	0.49	0.26	62.5
AOPP (µmol/L)	0.76	0.52-1	0.04	118.5	87.5
**Muscle damage**					
Creatine kinase (U/L)	0.73	0.57–0.89	0.012	98.55	77.78
Creatine kinase – myocardial band (ng/mL)	0.77	0.56–0.99	0.027	50.60	70
Troponin I (ng/mL)	0.68	0.52–0.84	0.029	0.207	75
Lactate (mmol/L)	0.69	0.51–0.87	0.042	10.50	70.59
Lactate dehydrogenase (µkat/L)	0.87	0.76–0.98	0.001	55.15	82.35
Aspartate aminotransferase (U/L)	0.84	0.72–0.97	0.003	160.7	81.25
Alanine aminotransferase (U/L)	0.78	0.59–0.98	0.023	17.85	88.89
**Sepsis**					
Procalcitonin (ng/mL)	0.86	0.64–0.96	0.01	2940	75
Aldolase (U/L)	0.79	0.62–0.97	0.014	18.35	88.89
**Immune system**					
Adenosine deaminase (U/L)	0.92	0.87-1	< 0.001	7680	92.96

ITIH4: inter-alpha trypsin inhibitor 4; FRAS: ferric reducing ability of saliva; AOPP: advanced oxidative protein products

### Correlation between analytes and the severity of the disease

The grade of severity of the disease showed a positive correlation with total protein (r = 0.75, p = 0.002), ALT (r = 0.62, p = 0.010), aldolase (r = 0.59, p = 0.040), and AOPP (r = 0.56, p = 0.040) values.

## Discussion

The results of this report indicate that there is an increase in the values of several salivary analytes related to stress, inflammation, redox status, and muscle damage in pigs infected with *S. suis* infection. In this study, aldolase and PCT, as well as ADA, were higher in the saliva of pigs with the infection compared to healthy pigs. These results are in line with previous reports in which these analytes were shown to be increased in the saliva of pigs with meningitis due to *S. suis* infection [8–10] and corroborate that this disease produces changes in biomarkers of sepsis (PCT and aldolase) and immune system (ADA) in saliva.

In the group of analytes related to stress, cortisol, sAA and OXT were higher in pigs with meningitis than in healthy ones. No previous report was found about changes in cortisol in any species due to *S. suis* infection, however, in fish, *Streptococcus iniae* produces increases in this analyte [13]. In general, it has been described that infectious diseases increase cortisol, especially free cortisol [14]. In accordance, the measurement of cortisol in saliva could have the advantage of reflecting free cortisol in comparison with plasma [5]. sAA increases in immune-mediated diseases in humans such as systemic lupus erythematosus, possibly due to stress associated with the disease and the immune system activation [15]. The increase in this enzyme in our study could be associated with the pain induced by the disease, as described in other diseases in pigs that can produce an increase in stress markers because of the pain and discomfort [16]. OXT is a nonapeptide hormone used to assess positive and stressful situations in animals [17, 18]. However, few studies evaluated its relationship with sepsis, despite a relationship between OXT and sepsis or other inflammatory states has been suggested [19]. OXT has been shown to have anti-inflammatory effects [20] and a possible protective role against sepsis [21] by limiting organ damage associated with this pathology [19, 21]. Thus, the increase in OXT concentrations in pigs with meningitis found in this report, in comparison to healthy pigs, could reflect this role.

Among the analytes associated with inflammation, Hp, ITIH4, and total protein concentrations increased in pigs affected by *S. suis*. Hp was 1.5 -fold higher, which is consistent with the performance of haptoglobin as a moderate acute phase protein (APP) in swine [22, 23]. Increased Hp has been previously described in the serum of pigs with *S. suis* [8]. In addition, this APP was found to increase in inflammatory and infectious processes such as the administration of lipopolysaccharide from bacteria (LPS) [24] and viral infections [25]. Increases of ITIH4, also known as pig-MAP, in serum have been described in different inflammatory conditions in the pig [26]. The increments of total protein found in the saliva of pigs with meningitis found in this study could be due to the higher protein production in inflammation states; as it was observed in the serum of pigs with bacterial infections due to the increases in several APPs [27].

In this report, the oxidant AOPP increased 1.5-fold in the saliva of pigs affected by *S. suis*. In a recent study, increased concentrations of salivary AOPP were observed in pigs with experimentally induced sepsis, potentially attributed to the release of oxidant products during the sepsis process [28].

The muscle marker CK was also increased in the animals with the *S. suis* infection. Increases in CK and AST in serum have been observed during transportation, mainly associated with the physical activity that accompanies the handling that can cause muscle damage in the animals [29]. The main source of serum CK is skeletal muscle, whereas AST or ALT are less specific for skeletal muscle than CK because of its presence also in the liver [30]. Therefore, increases in AST and ALT could also indicate the possible presence of liver damage associated with meningitis.

Troponin I concentration and LDH activity were also increased in the saliva of pigs with meningitis. Troponins are considered high-specific cardiac alteration markers in humans [30] and different animal species such as sheep [31], being previously related to myocarditis associated with infection by *Streptococcus* [32] or meningitis [33]; whereas increases in LDH can be produced in addition to cardiac damage by the injury of other muscle types. The findings of this study would be in line with a previous report in which increases in salivary metavinculin (VCL), a cytoskeletal protein that anchors actin to the cell membrane present, among other tissues, in the cardiac muscle was described in *S. suis* infection in pigs [34]. In this regard, it was reported that the amelioration of LPS-induced sepsis caused a reduction of LDH [35] and troponins [36] due to the reduction of inflammation in the cardiac tissues. The presence of myocardial damage suggested by the increments in troponin I and LDH was also supported by the increments in CK-MB observed in the saliva of pigs with meningitis of our study. CK-MB is mostly found in the myocardium and is a diagnostic marker for myocardial damage caused by viral [37] and bacterial infections [38]. Overall, it could be hypothesized that meningitis in pigs caused disturbances in cardiac muscle and these changes can be observed in saliva.

S100A8-A9 and S100A12 are calcium-binding proteins of the S100 family, located in the cytosol of neutrophils and/or monocytes [39]. They are released after the activation of these cells, and they are involved in inflammation and sepsis having an antimicrobial function [40, 41]. In humans, serum S100A8-A9 is considered a marker of inflammation that can increase in conditions such as severe traumatic brain injury [11] and sepsis [42] and in saliva increased in immune-mediated diseases [43]. In pigs, S100A8-A9 in saliva was increased in sepsis and stressful situations [44]. It is interesting to point out that both proteins showed a significantly high correlation in this study (Spearman r = 0.90, p < 0.0001), similar to what has been described in the serum of humans where a Spearman r of 0.87 with a p < 0.0001 was found between S100A8-A9 and S100A12 in a study that includes septic and healthy individuals [45]. Further studies should elucidate the reason for the increase of these two proteins in pigs infected with *S. suis.*

sAA, total proteins, ADA, S100A8-A9, S100A12, LDH, PCT, and AST presented values of area under the curve (AUC) > 0.8 at receiving operating characteristics (ROC) curve analysis and therefore a good ability to distinguish between healthy pigs and pigs with *S. suis* infection. However, it should be stressed that in most cases there was an overlap of the values of the analytes between both groups and due to the non-specific nature of these markers, they cannot be diagnostics of the disease.

The grade of severity of diseased animals showed a positive correlation with some selected analytes, having the highest correlation with total proteins. Although further studies should be carried out to elucidate the reason behind this correlation, the higher severity of the diseased animals could reflect a higher inflammation associated with increases in this analyte.

It is important to point out, as was previously indicated above, that the analytes studied in this report cannot be used for the diagnosis of *S. suis* infection since specific methods for its detection are needed. However, they can potentially be useful for some practical applications already. For example, some of these analytes, such as the APPs, could be biomarkers of early detection to predict the possible presence of the disease in the near future in non-symptomatic animals. In addition, they could be useful for monitoring treatment efficacy. Also, the biomarkers of sepsis such as PCT and aldolase could help in order to detect if there is a need for antibiotic use, as occur in humans [46]. This would be in line with the new veterinary medicine regulation in the EU moving from in-feed antibiotic treatment to more targeted approaches like individual injectable antibiotics, which reduce their use. In addition, it would be of interest to study the possible measurement of these analytes in ropes, which can assess in a general way the status of the pigs in a pen and be of easier practical use.

There are some limitations in the study that should be stated. First, only male pigs were recruited, therefore the gender of the animals has not been considered and the effects of sex in the analytes evaluated could not be investigated. In addition, although, the statistical power obtained in this report was higher than 0.8, indicating that from a statistical point of view the number of animals included in this study was adequate, it would be interesting to evaluate larger populations and also different farms. In these studies, ROC analysis to evaluate the sensitivity and specificity of different analytes alone or in combination should be made. It would also be interesting to evaluate the potential of these analytes to distinguish pigs with meningitis from pigs with other diseases and not only with healthy animals. Also, these additional studies should explore the possible application of these markers to detect early infection and monitor the disease.

## Conclusion

Overall, it can be concluded that the infection by *S. suis* produces changes in analytes related to sepsis such as PCT and aldolase, immune system such as ADA, stress such as sAA and OXT, inflammation such as Hp, ITIH4, total protein, S100A8-A9 and S100A12, redox status such as AOPP and muscle damage such as CK, CK-MB, AST, ALT, lactate, LDH, and troponin-I. These changes reflect that different pathophysiological mechanisms such as immune activation, stress, inflammation, oxidative disturbances or muscle damage are involved in this disease. In addition to provide information about the changes that occur in analytes in saliva in *S. suis* infection, this report opens the opportunity to further investigate these analytes as potential biomarkers of this disease in different situations such as early detection, prognosis or for monitoring response to treatment.

## Methods

A total of 56 growing male pigs [(*Sus scrofa domesticus*) (Landrace × Large White)] from 6 to 9 weeks old from a commercial farm, were used in this study. Pigs were placed in pens containing a standard feeder and a nipple drinker to provide ad libitum access to feed and water with a minimum space of 0.65 m^2^ per animal (Council Directive 2001/88/CE of 23 October 2001 amending Directive 91/630/CEE concerning minimum standards for the protection of pigs) and an average temperature of 24 ± 2 °C. The pig commercial farm was selected based on the criteria of having a history of *S. suis* outbreaks.

The animals were classified into two different groups: (A) the healthy group (n = 28), integrated by clinically healthy pigs, and (B) the meningitis group (n = 28), composed of pigs diagnosed with meningitis induced by *S. suis*. The criteria to select the pigs of the meningitis group were: (1) having clinical signs compatible with *S. suis* infection, (2) not having been treated before, and (3) being positive for *S. suis* at the bacterial isolation and characterization. For this purpose, blood samples were incubated on Columbia blood agar plates (Oxoid Ltd., Madrid, Spain) supplemented with 5% defibrinated pig blood for 48 h at 37ºC under aerobic conditions [47]. The isolates were identified using standard procedures and confirmed by a polymerase chain reaction (PCR) based on the glutamate dehydrogenase gene, as previously described [8]. All animals of the meningitis group tested positive for *S. suis* serotype 9. The pigs were treated after diagnosis with injectable amoxicillin (Unicillin, Univet Ltd). The pig farms had no metaphylactic treatment in place at this time because they are looking for new approaches, in compliance with the new EU veterinary medicine regulation.

The severity of the disease was classified on a five-point scale [1- No clinical signs (control group), 2- mild, 3- moderate, 4- high, 5- severe disease] based on the presence of hyperthermia, arthritis, and two grades of neurological signs (Table 3). Pigs were clinically examined by the farm veterinarian before sample collection.

**Table 3:** Severity scale. - No presence; + Moderate; ++ Severe

Grade of severity	Description	Hyperthermia	Arthritis	Neurological manifestations
1	No clinical signs	-	-	-
2	Mild disease	-	-	+
3	Moderate	+	-	+
4	High	+	+	+
5	Severe disease	+	+	++

In neurological signs, +: presence of mild to moderate ataxia or depression, ++: presence of severe sings such as convulsions, paddling, shivering, seizures, rolling eyes and/or head tilts

### Sampling

Saliva was obtained using a sponge attached to a flexible thin metal rod as previously described [10]. Samples were stored in ice during sampling and transportation to the laboratory where they were centrifuged (3000 g, 10 min, 4 °C) to collect the supernatants and stored at -80 °C before biochemical analysis. Additionally, blood samples were obtained from a jugular vein after saliva collection to perform bacterial isolation. The animal study protocol was approved by the Ethical Committee on Animal Experimentation (CEEA) of the University of Murcia (CEEA 563/2019).

### Biochemical analysis of saliva

#### Biomarkers of stress

Cortisol and OXT concentrations were measured by two amplified luminescent proximity homogeneous (AlphaLisa) assays previously validated in pig saliva samples [48, 49]. The sAA activity was measured by a commercial assay (a-Amylase, OSR6182, Beckman Coulter) previously validated in porcine saliva [50] in an Olympus AU400 autoanalyzer (Beckman Olympus AU400 Chemistry Analyzer, Beckman Coulter).

#### Biomarkers of inflammation

Hp concentration was measured by an assay based on AlphaLisa technology previously used in pig saliva [51]. ITIH4 was measured using a porcine species-specific commercially available ELISA kit (Porcine ITIH4, ElabSciences). The kit showed values of intra and inter-imprecision lower than 15% and accurate results in linearity under dilution for quantifying the ITIH4 in the saliva of pigs. Total protein concentration was analyzed using a colorimetric kit to measure Low-Complexity Region (LCR) proteins (protein in urine and CSF, Spinreact). S100A8-A9 was analyzed with a commercially available immunoturbidimetric assay (BÜHLMANN fCal Turbo® assay, Laboratories AG) using an Olympus AU400 autoanalyzer. This assay is designed for humans, but it has been demonstrated to have cross-reactivity with CALPS100A8-A9 in pigs [38] and it has been validated in the saliva of this species [44]. SA100A12 was measured with the SEB080Po Cloud-Clone ELISA kit, which is a specific assay for the measurement of this protein in pigs. This assay provided an intra and inter-assay imprecision lower then 15% and was linear after serial sample dilutions. 

### Biomarkers of redox status

FRAS was determined by the reduction of ferric-tripyridyltriazine (Fe3+-TPTZ, Sigma-Aldrich Co.) to the ferrous (Fe2+) form [52] and the AOPP based on a method previously described [53] and both were previously used in pig saliva [51]. These analyses were carried out using an Olympus AU400 autoanalyzer.

### Biomarkers of muscle damage

CK, CK-MB, AST, ALT, and lactate were measured using commercial kits from Beckman (Beckman Coulter Inc., Fullerton, CA, USA) while LDH was a commercial kit from Biosystem (Biosystem S.A., Barcelona, Spain) that was previously validated in the saliva of pigs [54]. These methods were performed using an Olympus AU400 autoanalyzer. Troponin I was quantified by a solid-phase enzyme-labeled chemiluminescent immunometric assay (IMMULITE Troponins I, Siemens Medical Solutions Diagnostics) in an IMMULITE 1000 immunoassay system.

CK, AST, ALT, LDH, lactate, and troponin I provided an intra- and inter-assay imprecision of < 15% and accurate results in linearity under dilution assays. In the case of troponin I determinations, values lower than the low limit of analytical detection of 0.2 ng/mL were established as 0.2 ng/mL.

### Biomarkers of the immune system

ADA was analyzed with a commercially available spectrophotometric automated assay (Adenosine Deaminase assay kit, Diazyme Laboratories), previously validated for porcine saliva [55].

### Biomarkers of sepsis

PCT was measured using an in-house indirect competitive AlphaLisa previously validated [10] and aldolase through a commercially available reagent kit (Aldolase, Randox Laboratories Ltd., Crumlin, UK) in an Olympus AU400 autoanalyzer.

### Statistical analysis

Data were assessed for normality by the Shapiro-Wilk method and showed non-normal distribution. A non-parametric approach was followed to analyze all the results. Mann-Whitney test was used to compare the differences in each variable between groups. Results were expressed as median and range. ROC curve was calculated for each variable to determine the cut-off that distinguished between groups. Those analytes showing a significant AUC were selected to calculate the cut-off values according to a protocol previously published [56]. A correlation study between the severity of the disease and the different analytes evaluated was performed through the Spearman test for non-parametric data. Results were considered significant when p < 0.05. The statistical analysis was performed using GraphPad Prism 9 software for Mac (GraphPad Software, LLC, San Diego, CA, USA). The statistical power of the results (1 – β) was calculated by a posthoc analysis by the G-Power program (Dusseldorf, Germany).

## Data Availability

Not applicable.

## Abbreviations

S. suis: Streptoccocus suis
CK: Creatine kinase
CK-MB: Creatine kinase myocardial band
LDH: Lactate dehydrogenase
ADA: Adenosine deaminase
PCT: Procalcitonin
SAA: Alpha-amylase
OXT: Oxytocin
AOPP: Advanced oxidation protein products
AST: Aspartate aminotransferase
ALT: Alanine aminotransferase
CALP: Calprotectin
FRAS: Ferric reducing ability of saliva
LPS: Lipopolysaccharide
AUC: Area under the curve
ROC: Receiving operating characteristics
EU: European Union
PCR: Polymerase chain reaction
CEEA: Ethical Committee on Animal Experimentation
LCR: Low-Complexity Region
ITIH4: Inter-alpha trypsin inhibitor 4
